# Graphical user interface design to improve understanding of the patient-reported outcome symptom response

**DOI:** 10.1371/journal.pone.0278465

**Published:** 2023-01-24

**Authors:** Mangyeong Lee, Danbee Kang, Yeongrae Joi, Junghee Yoon, Youngha Kim, Jinhwang Kim, Minwoong Kang, Dongryul Oh, Soo-Yong Shin, Juhee Cho

**Affiliations:** 1 Center for Clinical Epidemiology, Samsung Medical Center, Seoul, Korea; 2 Department of Digital Health, SAIHST, Sungkyunkwan University, Seoul, Korea; 3 Department of Clinical Research Design and Evaluation, SAIHST, Sungkyunkwan University, Seoul, Korea; 4 Department of Radiation Oncology, Samsung Medical Center, Sungkyunkwan University School of Medicine, Seoul, Korea; La Trobe University - Melbourne Campus: La Trobe University, AUSTRALIA

## Abstract

**Background:**

Symptom monitoring application (SMA) has clinical benefits to cancer patients but patients experience difficulties in using it. Few studies have identified which types of graphical user interface (GUI) are preferred by cancer patients for using the SMA.

**Methods:**

This is a cross-sectional study aimed to identify preferred GUI among cancer patients to use SMA. Total of 199 patients were asked to evaluate 8 types of GUIs combining text, icon, illustration, and colors using mixed-methods. Subgroup analyses were performed according to age and gender.

**Results:**

The mean age of the patients was 57 and 42.5% was male. The most preferred GUI was “Text + Icon + Color” (mean = 4.43), followed by “Text + Icon” (mean = 4.39). Older patients (≥ 60 years) preferred “Text + Icon” than younger patients (*p* for interaction < 0.01). Simple and intuitive text and icons were the most useful GUI for cancer patients to use the SMA.

**Conclusion:**

Simple and intuitive text and icons were the most useful GUI for cancer patients to use the SMA. Researchers need to be careful when applying realistic face drawings to cancer symptom monitoring applications because they can recall negative images of cancer.

## Introduction

Cancer is a leading cause of death worldwide, and an estimated 19.3 million new cancer cases occurred in 2020 [1]. Cancer patients experience a wide range of physical and psychological symptoms owing to anticancer therapies or the disease itself [2, 3]. Acute symptoms, especially those related to anticancer treatment, typically manifest during the interim period, and waiting to capture these during clinic visits might cause peak symptom distress to be missed. However, poorly controlled symptoms can lead to unplanned clinic or emergency department visits and unplanned hospitalizations [4–7]. In addition, a recent clinical trial demonstrated that active symptom management using mobile technology has contributed to improved survival rates [8, 9]. Therefore, interest in monitoring systems that use patient-reported symptoms through mobile devices has been increasing [10].

There have been several mobile symptom monitoring applications (SMA) that can facilitate the timely detection of patients’ symptoms [9, 11–14]; however, patients with low digital health literacy have difficulties in effectively utilizing and interacting with technologies [15–17]. To resolve this problem, the U.S. Food and Drug Administration recommends applying human factors that identify and address end-user requirements, mobile system design, and functionality to users’ capabilities, needs, and expectations in the development process for digital health services [18–21]. Among these human factors, graphical user interfaces (GUIs), such as the layout, font style, interaction element, and task scenario could affect the patients’ usability for SMAs [11, 22]. GUI is a form of user interface that allows users to interact with electronic devices through graphical icons and visual indicators, instead of text-based interfaces [23]. The elements on the screen, such as color, size, and design, affect user satisfaction and understanding [24].

Previous studies have shown that GUIs are strongly preferred over text-based interfaces for novice and low-literacy users [25], and the extensive use of graphics is recommended for low-literacy communities [26]. Several studies related to interface design were conducted regarding questionnaire layout, statement expression, position of image, or color brightness [27–29]. Still, few studies have evaluated different GUI designs of response scales for patients’ understanding and preferences [20, 30–33]. This study aims to identify preferred GUI among cancer patients to use SMA.

## Methods

### GUI design

“Vomiting” was chosen to evaluate which GUI is the most helpful in understanding symptoms, because it is one of the most common symptoms in cancer patients [34]. The symptoms (vomiting) were described on a 5-point Likert scale (0 = none, 1 = mild, 2 = moderate, 3 = severe, and 4 = very severe).

**Fig 1:**
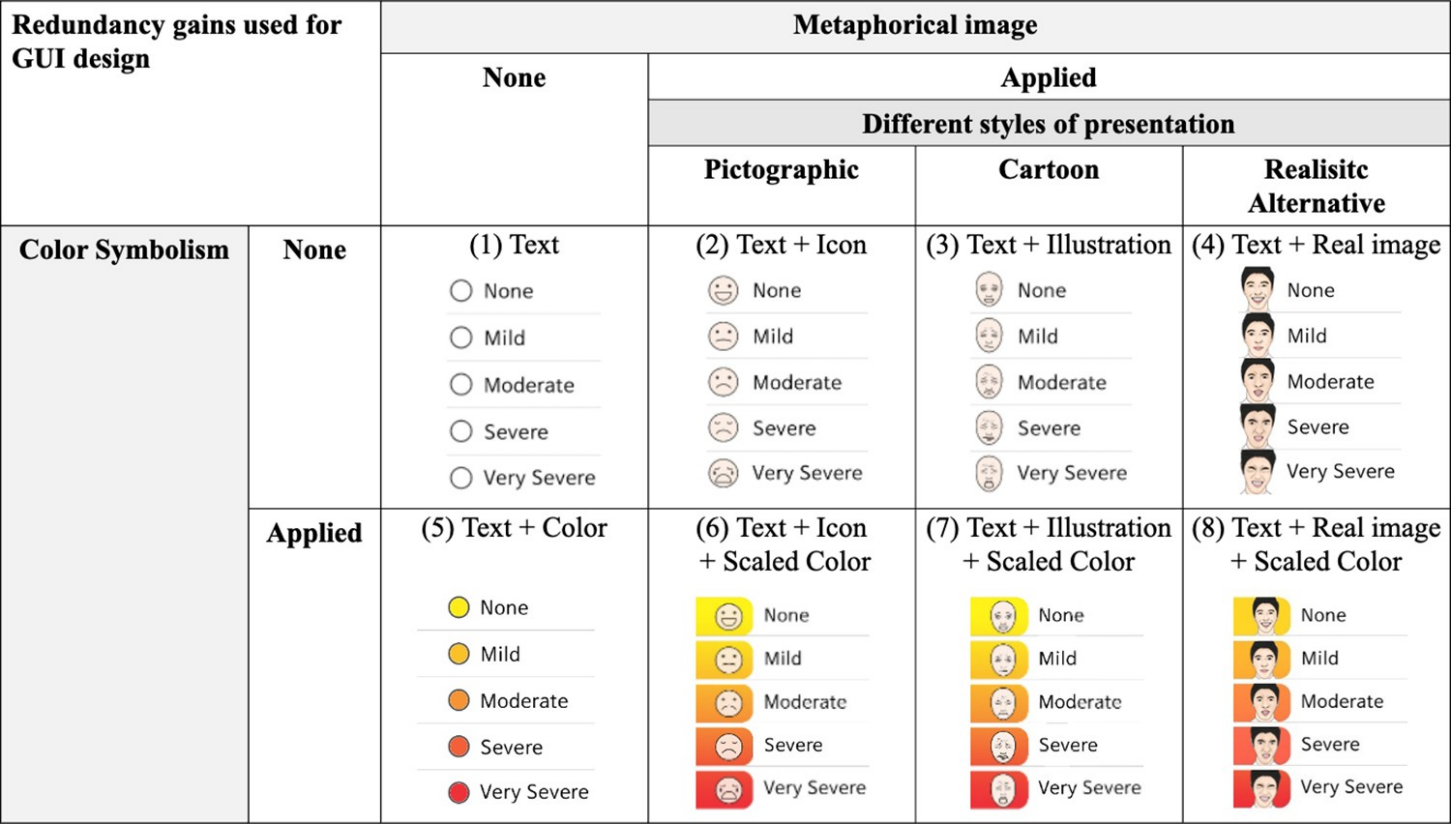
Graphical user interface (GUI) design to help understand the symptoms.

Based on a previous study that compared five styles of visuals to examine one that makes information increasingly accessible to patients [35], eight types of GUIs were developed by combining texts, icons, illustrations, and colors, as shown in Fig 1: 1) “Text only”; 2) “Text and Icon”; 3) “Text and Illustration”; 4) “Text and Real image”; 5) “Text and Scaled color”; 6) “Text, Icon, and Scaled color”; 7) “Text, Illustration, and Scaled color”; and 8) “Text, Real image, and Scale color”. In the design, we considered ‘*redundancy gain*’ which can evoke responses more quickly and accurately by providing separate stimuli redundantly such as color and shape [36]. In terms of shape, we applied metaphorical images implying the meaning of each response scale, with different styles of the presentation [37]. The “Real image” consists of original face photos to represent the given condition of cancer patients with facial expressions [38]. “Illustration” is a simple line drawing based on face photos. Icon represents the pictorial symptom scales, because the smiley is well known as an effective visual scale tool, regardless of a patient’s literacy or language skills [39]. In addition, we included color symbolism to help patients perceive symptoms, as in previous studies [40, 41]. The red and yellow colors were chosen. Red is often associated with negative emotions, such as danger, stop [42, 43], or mistakes [44]. Yellow is often associated with positive emotions, such as cheerful [45, 46]. These colors are also commonly used in health apps [47, 48].

The eight types of GUIs were designed by a head and neck oncologist, a behavior scientist, two cancer educators, and a visual graphic designer. The GUIs were refined using iterative design testing, modification, and retesting processes. The designed GUIs were tested on a tablet with full HD resolution (1920px × 1200px) (Samsung Galaxy Tab A).

### Study design and participants

A cross-sectional mixed-methods study was conducted of cancer patients attending the outpatient clinic at the Samsung Medical Center in Seoul, Korea, from April 2017 to September 2017. Subjects were eligible to participate in the study if they were 18 years of age or older, had a histologically confirmed diagnosis of cancer, had no evidence of physical or psychological problems at the time of the survey, and were able to read Korean.

**Fig 2:**
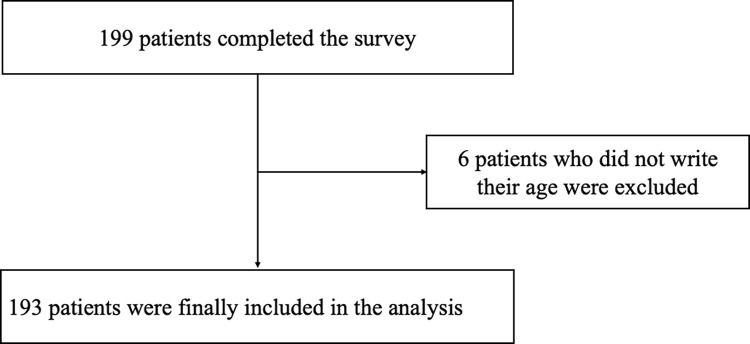
Study design and participant flow.

A total of 199 patients agreed to participate in the study. Among them, people who had no information on age (N = 6) were excluded from the study. The final study group included 193 patients with cancer, as shown in Fig 2. The study was approved by the Institutional Review Board of Samsung Medical Center, and all patients provided written informed consent (No. SMC-2015-12-011).

### Measurement

A cross-sectional study involving both quantitative and qualitative methods was conducted by four trained researchers. For the quantitative phase, a tablet PC containing examples of each GUI was provided so that participants could answer questions based on their understanding of each type of GUI. Participants were also asked to choose the most helpful GUI for understanding the symptoms among the eight types of GUIs. Information regarding gender, age, highest completed level of education, type of cancer, income level, time since cancer diagnosis, and current treatment status were collected using a standardized questionnaire.

After the quantitative survey, the patients were asked to participate in the qualitative phase. If the patients agreed to participate, they were interviewed based on the questionnaires submitted. A total of eight patients agreed to participate in the survey, and the interviewer asked the patients the reasons they preferred or did not prefer a type of GUI. The interview took 10–20 min per patient (S1 Table).

### Statistical methods

Descriptive statistics were used to report the characteristics of the participants and the mean and standard deviation (SD) score of each GUI. We used mixed-effect models for the repeated measurement of data in the eight types of GUIs. The mean differences and 95% CIs were calculated using a linear mixed model. Interaction analysis was used to evaluate whether the association of the type of GUI with understanding scores differed by age group (< 60 and ≥ 60 years). In South Korea, 55% of 25–64 year-olds have a higher level of educational attainment than older adults whose age is 55–64 years [49]. Therefore, older adults, in their 60s or older, were set as the low-literacy population. In addition, we compared the effects of GUI by gender.

All reported *P*-values were two-sided, and the significance level was set as 0.05. All analyses were performed using STATA version 14 (StataCorp LP, College Station, TX, USA).

## Results

### Characteristics of the study population

The mean age (SD) of the participants was 57.0 ± 10.9 years, and 42.5% were male (Table 1). In terms of clinical characteristics, 70% of the patients were under active treatment, and the median time since cancer diagnosis was one year (range of 0–12 years).

**Table 1:** Characteristics of the study population.

		Participants
		(N = 193)
		N (%)
**Age (years), Mean (SD)**		57.0 (10.9)
	< 60	119 (61.7)
	≥ 60	74 (38.3)
**Sex**		
	Male	82 (42.5)
	Female	111 (57.5)
**Educational level**		
	Less than College	62 (32.1)
	College or higher	120 (62.2)
	Unknown	11 (5.7)
**Income level (USD)**		
	< 2,000	25 (13.0)
	2,000≤ –<4,000	37 (19.2)
	≥ 4,000	99 (51.3)
	Unknown	32 (16.6)
**Type of cancer**		
	Breast cancer	77 (39.9)
	Gastric cancer	43 (22.3)
	Lung/esophagus cancer	26 (13.5)
	Blood cancer	21 (10.9)
	Prostate cancer	11 (5.7)
	Head and neck cancer/Brain tumor	6 (3.1)
	Gynecologic cancer	2 (1.0)
	Other cancer	7 (3.6)
**Time since cancer diagnosis (years), Mean (SD)**		1.9 (2.6)
	0 years	76 (39.4)
	1 to 2 years	67 (34.7)
	3 to 4 years	16 (8.3)
	≥ 5 years	30 (15.5)
	Unknown	4 (2.1)
**Current treatment** ^**a**^		135 (70.0)
	Yes	135 (70.0)
	No	57 (30.0)
**Type of treatment (n = 135)**		
	Operation	12 (8.9)
	Chemotherapy	89 (65.9)
	Radiotherapy	48 (35.6)
	Hormone therapy	5 (3.7)

SD = Standard Deviation; USD = United States Dollar

^a^ One value was missed

### Comparison of patient preferences according to graphical user interface (GUI) designs

**Fig 3:**
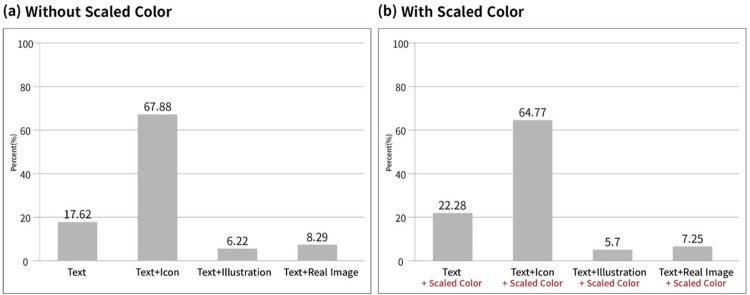
Proportion of most helpful to understand the symptoms among the graphical user interface (GUI) designs. (a) Without Scaled Color, (b) With Scaled Color.

As shown in Fig 3, most patients preferred “Text + Icon,” regardless of the presence (64.77%) or absence of (67.88%) scale colors.

In terms of scores for help to understand (Table 2), the highest understanding score was for “Text + Icon + Color” (mean = 4.43), followed by “Text + Icon” (mean = 4.39).

**Table 2:** Linear mixed linear regression model to compare the score of understanding of terms according to graphical user interface (GUI) design.

GUI*	Mean (SD)	Coefficient (95% CI)
**Text**	3.21 (1.08)	*Reference*
**Text + Color**	3.33 (1.14)	0.12 (-0.09, 0.33)
**Text + Icon**	4.39 (0.92)	**1.18 (0.97, 1.39)**
**Text + Icon + Color**	4.43 (0.89)	**1.22 (1.01, 1.44)**
**Text + Illustration**	2.14 (1.11)	-1.07 (-1.28, -0.85)
**Text + Illustration + Color**	2.17 (1.06)	-1.04 (-1.25, -0.83)
**Text + Real image**	2.29 (1.23)	-0.92 (-1.13, -0.70)
**Text + Real image + Color**	2.33 (1.14)	-0.88 (-1.09, -0.66)

* An example of a GUI is shown in Fig 1.

The lowest understanding score was for “Text + Illustration” (mean = 2.14). When we compared to “Text only,” although “Text + Icon” (Coefficient = 1.18; 95% confidence interval (CI) = 0.97, 1.39) and “Text + Icon+ Color” (Coefficient = 1.22; 95% CI = 1.01, 1.44) had significantly higher understanding scores, “Text + Illustration” (Coefficient = -1.07; 95% CI = -1.28, -0.85) and “Text + Real image” (Coefficient = -0.92; 95% CI = -1.13, -0.70) yielded significantly lower understanding scores, even with color (Table 2).

Although some patients reported that “Icon” was frivolous for severe symptoms, many patients preferred it because it may enhance the understanding of the symptom scale and is a familiar GUI. Otherwise, patients felt emotional discomfort when viewing illustrations or real images, because these images reminded them of how sick they were and distressed them (see S1 Table). In addition, patients reported that it was difficult to intuitively recognize scale changes in illustrations and real images. Patients also mentioned that scaled colors helped them recognize the difference between mild and severe conditions more clearly.

### Sex and age differences in the preferences

While the preferences were not different between males and females, there was a difference according to the age group. A significantly higher score for “Text + Icon” was consistently observed in all age groups and genders. As shown in Table 3, participants aged ≥ 60 years had a higher score than younger participants (*p* for interaction < 0.01). Younger patients had a lower score in “Text + Real image” than older patients (*p* for interaction < 0.01).

**Table 3:** Subgroup analysis for multivariable mixed linear regression to compare score of understanding of terms according to graphical user interface (GUI) design.

	By age			By sex		
GUI*	< 60 (N = 119) Coefficient (95% CI)	≥ 60 (N = 74) Coefficient (95% CI)	P for interaction	Male (N = 82) Coefficient (95% CI)	Female (N = 111) Coefficient (95% CI)	P for interaction
**Text**	*Reference*	*Reference*		*Reference*	*Reference*	
**Text + Color**	0.09 (-0.18, 0.36)	0.16 (-0.18, 0.5)	0.25	0.12 (-0.2, 0.45)	0.12 (-0.16, 0.4)	0.86
**Text + Icon**	**1.08 (0.82, 1.35)**	**1.34 (1.00, 1.68)**	**< 0.01**	**1.16 (0.83, 1.48)**	**1.20 (0.92, 1.48)**	0.19
**Text + Icon + Color**	**1.16 (0.89, 1.43)**	**1.32 (0.98, 1.67)**	0.28	**1.10 (0.77, 1.42)**	**1.32 (1.04, 1.59)**	0.08
**Text + Illustration**	-1.33 (-1.60, -1.06)	-0.65 (-0.99, -0.31)	0.75	-0.90 (-1.23, -0.58)	-1.19 (-1.47, -0.91)	0.98
**Text + Illustration + Color**	-1.29 (-1.56, -1.03)	-0.64 (-0.98, -0.29)	0.46	-0.85 (-1.18, -0.53)	-1.18 (-1.46, -0.90)	0.32
**Text + Real image**	-1.01 (-1.28, -0.74)	-0.77 (-1.11, -0.43)	**< 0.01**	-1.13 (-1.46, -0.81)	-0.76 (-1.04, -0.48)	0.13
**Text + Real image + Color**	-1.09 (-1.36, -0.82)	-0.53 (-0.87, -0.19)	**0.01**	-0.95 (-1.28, -0.63)	-0.82 (-1.1, -0.54)	0.55

* An example of a GUI is shown in **Fig 1**.

## Discussion

In this study, “Text + Icon + Color” was the most preferred GUI for improving understanding of their symptoms in cancer patients. Cancer patients also responded that “Text + Icon” was helpful to understand the severity of their symptoms even without scaled color. On the other hand, “Illustration” or “Real image” was associated with a lower understanding score. Especially, older patients preferred “Icon” compared to younger patients.

In this study, “Text + Icon + Color” was the most preferred GUI for improving understanding of their symptoms. As in previous studies [40, 41], colors increased the perception of symptoms experienced when compared with the non-color condition. However, when we compared understanding score of patients according to the color, and the differences were not statistically significant. “Icon” had a much greater impact on the patients’ preferences. Regardless of scaled color, “Icon” was preferred by the patients. A study regarding interface design of multimedia learning presented that shape with neutral color (grayscale) was most effective compared to the shape combined with color in learning process [50]. It might be due to that “Icon” which is based on pictographic presentation delivers more intuitive and concrete information than color symbolism.

On the other hands, illustration or real image was associated with lower understand score. As in a previous study [51], the detailed parts made patients feel redundant and did not provide additional information. In addition, cognitive overload can occur when the visuals contain too much demand [52]. ‘Cognitive Load Theory’ basically assumes that the human cognitive system has a limited working memory capacity and humans may feel difficult to take a task if cognitive efforts for the task exceed their limits [53]. In addition, some patients reported psychological repulsion as it directly reminded them that they had lost hair and how sick they were in the qualitative interview. Patients’ experience had a negative perception for “Illustration” or “Real image,” as it seemed to have cancer stigma [54]. According to a study with the public, strong identification with celebrity cancer death through the mass media was associated with negative emotional reactions, resulting in stigma-related perceptions [55]. Thus, in our findings, cancer patients might experience negative feelings from identification with the displayed images reminiscent of pain and hair loss. This result suggests that while providing GUIs to cancer patients, cancer stigma should be considered.

In subgroup analysis, older patients preferred “Icon” to “Text only” more often than younger patients. Regarding the impact of scaled colors, younger (Coefficient = 1.16; 95% CI = -0.89, 1.43) and female (Coefficient = 1.32; 95% CI = -1.04, -1.59) participants exhibited a higher score for help to understand than older (Coefficient = 1.32; 95% CI = 0.98, 1.67) and male (Coefficient = 1.10; 95% CI = 0.77, 1.42) participants when “Icon” and colors were used together. Previous studies have also shown that older and male individuals are color deficient compared to younger and female individuals [56–58]. The simple shape of the GUI helps to understand health information in low-literacy patients [59–61]. Previous research has shown that low-literate people are more easily distracted by redundant details than well-literate people, which would explain our findings [51, 62]. Based on the recent education level of Koreans [49, 63], age could be considered a potential indicator of low literacy. Although well-designed visuals could have a positive effect on how information is perceived, not all visuals are effective because literate and low-literate people differ in perception preference [64, 65].

This study had several limitations. First, there is no standard tool for measuring the understanding of GUIs in patients with cancer. Thus, the survey used in this study does not prove its validity or reliability. Second, preference was measured using only a single symptom, vomiting. Therefore, our results may not be generalizable to other types of cancer symptoms or patients with different diseases. Third, we did not measure the patients’ literacy level. Fourth, cancer patients were recruited from a hospital located in the Seoul metropolitan area. Accordingly, future research should be conducted using different patient samples.

In conclusion, simple and intuitive text and icon was the most useful GUI for cancer patients to report their symptoms. This study could improve the GUI design guidelines for mobile healthcare applications (apps) for cancer patients. Design recommendations and useful features that can be applied during mobile app design for cancer patients were identified. The results of this study demonstrated how the potential use and acceptance of different types of GUI features could be influenced not only by the usefulness of specific functions but also by the current context of use. Further research is necessary to investigate the effect of GUI design with diverse patients and contexts.

## Supporting information

S1 Table Findings from qualitative interview. (DOCX)

## Abbreviations

ICT: Information and Communication Technology
SMA: Symptom Monitoring Applications
Apps: Applications
GUI: Graphic User Interface
Full HD: Full High Definition
Tab: Tablet PC
SD: Standard Deviation
CI: Confidence Intervals
