# Supplementary material for: Graphical user interface design to improve understanding of the patient-reported outcome symptom response
Source: PLoS One. 2023 Jan 24;18(1):e0278465. doi: 10.1371/journal.pone.0278465 (PMC9873161; doi:10.1371/journal.pone.0278465)
Supplement: S1 Table — (DOCX) [file pone.0278465.s001.docx]

**S1 Table.** **Findings from qualitative interview.**

| **Graphical user interface (GUI) design** | Advantages | Disadvantages |
| --- | --- | --- |
| **Metaphorical Image** |  |  |
| Text | The meaning of the symptoms scale is sufficiently understandable. | Visually looks empty compared to the answers with drawings included. |
| Text + Icon | Pictures help to enhance the understanding of the symptoms scale comprised only of words.  It is easy to look at due to familiarity from childhood.  Facial expressions and their changes are easily identifiable. | It seems as if it is a little light like cartoons to express the seriousness of the symptoms. |
| Text + Illustration | The realistic pictures seem to represent my current situation well. | It is rather uncomfortable to look at the frowning face.  The picture too directly shows cancer patients that have lost hairs.  It is uncomfortable to look at and be reminded of how sick the patients are.  It is difficult to see the facial expressions through the pictures, and it is difficult to see the changes. |
| Text + Real Image | The realistic pictures seem to represent my current situation well. | It is rather uncomfortable to look at the frowning face.  It is difficult to see the facial expressions through the pictures, and it is difficult to see the changes. |
|  |  | The real image is composed of only male. |
| **Color Symbolism** |  |  |
|  | The colors make it easy to clearly see the scale.  The colors added to the background of the pictures makes it clear to look at them.  It is easier to objectively understand the meaning of the colors when they are presented with a picture. | The meaning of the colors is abstract.  The various color tones make it look complex. I wish they were all uniformed in colors.  The combination of words, pictures, and colors make it look complex to look at.  I wish the color tone was a little lighter. The darker tone makes the visibility to be low.  I wish the answer “none” would be depicted in white. The yellow color does not really represent the meaning of “none” very well. |
